# Assembly and Analysis of the Genome Sequence of the Yeast *Brettanomyces naardenensis* CBS 7540

**DOI:** 10.3390/microorganisms7110489

**Published:** 2019-10-26

**Authors:** Ievgeniia A. Tiukova, Huifeng Jiang, Jacques Dainat, Marc P. Hoeppner, Henrik Lantz, Jure Piskur, Mats Sandgren, Jens Nielsen, Zhenglong Gu, Volkmar Passoth

**Affiliations:** 1 Department of Biology and Biological Engineering, Systems and Synthetic Biology, Chalmers University of Technology, SE-412 96 Göteborg, Sweden; nielsenj@chalmers.se; 2 Department of Molecular Sciences, Swedish University of Agricultural Sciences, Box 7015, SE-75007 Uppsala, Sweden; mats.sandgren@slu.se (M.S.); volkmar.passoth@slu.se (V.P.); 3 Key Laboratory of Systems Microbial Biotechnology, Tianjin Institute of Industrial Biotechnology, Chinese Academy of Sciences, Tianjin 300308, China; jiang_hf@tib.cas.cn; 4 Department of Medical Biochemistry and Microbiology, Uppsala University, Box 582, 752 37 Uppsala, Sweden; jacques.dainat@nbis.se (J.D.); m.hoeppner@ikmb.uni-kiel.de (M.P.H.); henrik.lantz@nbis.se (H.L.); 5 National Bioinformatics Infrastructure Sweden (NBIS), 752 37 Uppsala, Sweden; 6 Institute of Clinical Molecular Biology, Christian-Albrechts-University of Kiel, 24118 Kiel, Germany; 7 Department of Biology, Lund University, 223 62 Lund, Sweden; biol.bibl@biol.lu.se; 8 Division of Nutritional Sciences, Cornell University, Ithaca, NY 14853, USA; zg27@cornell.edu

**Keywords:** genome, yeast, *Brettanomyces naardenensis*

## Abstract

*Brettanomyces naardenensis* is a spoilage yeast with potential for biotechnological applications for production of innovative beverages with low alcohol content and high attenuation degree. Here, we present the first annotated genome of *B. naardenensis* CBS 7540. The genome of *B. naardenensis* CBS 7540 was assembled into 76 contigs, totaling 11,283,072 nucleotides. In total, 5168 protein-coding sequences were annotated. The study provides functional genome annotation, phylogenetic analysis, and discusses genetic determinants behind notable stress tolerance and biotechnological potential of *B. naardenensis*.

## 1. Introduction

Yeasts belonging to the genus *Brettanomyces* (teleomorph name *Dekkera*, which should, according to the principle “one fungus, one name”, no longer be used [1]) are of general interest, both as evolutionary models for the development of the fermentative lifestyle, as spoilage or production organisms in the generation of soft drinks, alcoholic beverages, and bioethanol production [2,3,4,5]. *Brettanomyces* yeasts have been firstly identified as spoilage organisms in beverages, but also as part of the natural microbial population in spontaneously fermented beers [2,6]. 

The acetate-producing, Crabtree-negative yeast *B. naardenensis* was previously shown to spoil carbonated beverages and soft drinks with pH ranging 2.6–3.2 [7,8]. The *B. naardenensis* strain CBS 7540 was isolated in 1990 from a soft drink in South Africa (Natal). Occurrence of *B. naardenensis* in Cabernet Sauvignon wine was also reported [9]. On the other hand, this yeast may also have some biotechnological potential. It was shown to be able to produce ethanol from xylose [10] and, unlike other species of the genus *Brettanomyces*, to assimilate soluble starch. The broad substrate range of *Brettanomyces* can contribute to reduction of residual sugar content in beverages production. This property can be used for the production of super-attenuated and lower-calorie beers [11]. The ability to assimilate an alternative nitrogen source, nitrate [12], may in part explain the growth of *Brettanomyces* in highly dosed hopped wort and also during beer maturation. Hops are a natural source of nitrate, which can be used as a nitrogen source by *Brettanomyces* species. in beer brewing, and thus result in their proliferation, at the same time reducing the nitrate content. Nitrate removal from drinks may decrease cancer and other health risks [13].

Negative health and economic consequences of alcohol consumption have awoken an interest in the production of non-alcoholic beverages by the brewing industry using non-conventional yeasts. Studies on *Brettanomyces* yeasts showed that this group of yeasts is not only a source for deterioration of the sensory qualities of drinks, but in some cases can also provide beneficial aromas for craft and specialty beverages [6]. The *Brettanomyces* aroma wheel was recently developed [14]. Ethyl acetate, lactate, hexanoate, and octanoate produced by *Brettanomyces* yeasts contributes to tropical fruit and pineapple-like flavors of lambic and gueuze beers. Esterification of cheesy-flavored long-chain fatty acids (C9, C10, C12, C14, C16) by *Brettanomyces* yeasts leads to generation of the grape-like beer flavor [15]. Beta-glucosidase activity of *Brettanomyces* contributes to generation of flavor-active aglycones, such as benzaldehyde, linalool, or eugenol from glucosides present in hops or fruits [16]. Yeasts can further convert monoterpenes into beta-citronellol or alpha-terpeniol, boosting the floral citrusy flavor of a beverage [17]. Beta-glucosidase activity of *Brettanomyces* was also explored for production of the antiaging compound, resveratrol [18]. 

Exploring the full biotechnological potential of *Brettanomyces* yeasts can be considerably facilitated by knowledge of the whole genome sequence. Up to now, mainly the genomes of strains of *Brettanomyces bruxellensis*—isolated from wine, lambic beer, and distilleries [19,20,21,22,23,24,25,26,27]—have been sequenced. The genomes of single strains of *Brettanomyces custersianus* and *Brettanomyces anomalus* are also available [28]. This study provides the first annotated genome of *B. naardenensis*. 

## 2. Materials and Methods

### 2.1. Cultivation

Two milliliters of *B. naardenensis* culture grown in 100 mL YPD at 25 °C to OD 10 were harvested. Aerobic batch cultivation of the strain for RNA isolation was performed using defined minimal media supplemented with 2% glucose in 1L Multifors fermenters (Infors HT, Bottmingen, Switzerland). 

### 2.2. Genome Sequencing

The genomic DNA of *B. naardenensis* CBS 7540 was prepared using a standard zymolyase and phenol-chloroform extraction. A sample quality test was made for DNA sample, and the qualified sample was used to construct libraries. Covaris or Bioruptor was used to break the genomic DNA into smaller fragments (less than or equal to 800 bps). T4 DNA Polymerase, Klenow DNA Polymerase, and T4 PNK were used for end repair of the small DNA fragments. Three prime end A addition and adapter ligation were performed using taq-polymerase. The target DNA fragments were size-selected by electrophoresis and enrichment was performed using PCR amplification and purification. The qualified library was cluster prepared and sequenced. Genome sequencing was performed using the Illumina GA II (Illumina, San Diego, CA, USA) by generating multiplexed paired-end libraries (an average insert size of 500 bp). Raw fluorescent images and call sequences was processed using a base-calling pipeline (Solexa Pipeline-version 1.0). 

### 2.3. RNA Library Preparation and Sequencing 

Isolation and processing of RNA from triplicates of batch cultures were performed as previously described [29]. The RNA was treated with the RiboMinus™ Eukaryote Kit (Thermo Fisher Scientific, Waltham, MA, USA) to remove ribosomal RNA and purified using Agencourt RNAClean XP Kit (Beckman Coulter, Indianapolis, IN, USA). The size and quantity of RNA fragments were assessed on the Agilent 2100 Bioanalyzer system (RNA 6000 Pico kit, Agilent, Santa Clara, CA, USA). Libraries were prepared using the Ion Total RNA-Seq kit for the AB Library Builder System (Life Technologies). Samples were then quantified using the Agilent 2100 Bioanalyzer system (High Sensitivity DNA kit, Agilent) and pooled followed by emulsion PCR on the Ion OneTouch™ 2 system using the Ion Proton™ Template OT2 200 v2 Kit (Life Technologies, Waltham, MA, USA) chemistry. Templated Ion Sphere particles were enriched using the Ion OneTouch™ ES (Life Technologies). Samples were loaded on a two Ion PI v2 Chips and sequenced on the Ion Proton™ System using Ion Proton™ Sequencing 200 v2 Kit (200 bp read length, Life Technologies) chemistry.

### 2.4. Genome Assembly

The raw data (fastq format) were cleaned by FastQC and fastx_toolkit. The read size was 250 × 2 bp. We used SOAPdenovo1.05 to assemble the cleaned reads [30]. All raw reads were mapped onto the scaffolds using SOAPaligner [31] to evaluate the single base accuracy of the assembled genome sequences. 

### 2.5. Transcriptome-Guided Genome Annotation

The annotation of the *B. naardenensis* CBS 7540 genome assembly was built in three steps: Prediction of candidate cDNAs using transcriptome data (assembled de-novo with Trinity and genome-guided with tophat and cufflinks) within the PASA package, followed by training of the SNAP ab-initio gene finder, and finally the actual gene build combining evidence alignments and ab-initio predictions using the Maker2 package. The annotation was deposited into European Nucleotide Archive (ENA) using EMBLmyGFF3 [32]. The annotated genome sequence of *B. naardenensis* CBS 7540 was deposited in the European Nucleotide Archive (ENA) with accession numbers CAACVR010000001 to CAACVR010000076 under project number PRJEB30032

### 2.6. Functional Genome Annotation

Functional genome annotation. Functional annotation of transcripts was performed using the Blast2Go pipeline (available online: http://www.blast2go.com/b2ghome), based on best BLAST matches of predicted cDNAs against the RefseqP fungal reference dataset. Annotation of *B. naardenensis* CBS 7540 was performed by searching the KOG (available online: http://www.ncbi.nlm.nih.gov/COG/) database using BLAST software.

### 2.7. SNP Analysis

To generate reads for analysis of variants, we resequenced the genome of *B. naardenensis* CBS 7540. The genomic DNA of *B. naardenensis* CBS 7540 was prepared using a ZYMO RESEARCH Quick DNA™Fungal/BacterialMiniprep KitCatalog No. D6005 according to manufacturer’s instructions. The input DNA was quantified using Qubit dsDNA HS Assay Kit (Invitrogen, Waltham, MA, USA) and the sample purity was determined using NanoDrop. Libraries with Illumina-compatible adapters were constructed using the KAPA HyperPlus Library Prep Kit (ROCHE, Basel, Switzerland) by following the instructions of the manufacturer. The finished libraries were quantified with Qubit dsDNA HS Assay Kit (Invitrogen) and the average library size was determined using DNF-473 Standard Sensitivity NGS Fragment Analysis Kit (1 bp–6000 bp); (Agilent). The generated multiplexed paired-end libraries had an average library size of 584 bp. Whole-genome sequencing was performed using the MiSeq Reagent Kit v2, 500 Cycles (Illumina) on the MiSeq sequencing platform (Illumina, San Diego, CA, USA) with 10 pM flow cell loading. The sequencing chemistry employed 4-channel sequencing-by-synthesis (SBS) technology. Reads generated by CBS 7540 genome resequencing were mapped to the genome assembly of *B. naardenensis* CBS 7540 by using BWA version 0.7.4 [33]. Identification of the various variants (SNP and indels) was performed as described earlier [27]. 

### 2.8. Phylogenetic Tree Construction

The proteomes of *Debaryomyces hansenii*, *Kluyveromyces lactis*, *Ogataea polymorpha*, *Pichia kudriavzevii*, *Yarrowia lipolytica*, *Komagataella pastoris*, *Candida albicans*, and *Saccharomyces cerevisiae* were downloaded from Ensembl and NCBI databases. We first built the orthologous groups of within 12 yeast genomes. Then, 2328 gene groups, which had a strict and phylogeny-based one-to-one orthology relationship in all species included in the phylome, were respectively aligned by ClustalOmega, and trimmed using trimAl (gap-score cutoff 0.5, conservation score 0.5), and then concatenated into a single alignment. Finally, a maximum likelihood tree was constructed by MEGA. All nodes received the highest support in terms of approximate likelihood ratio tests and of a bootstrap analysis of 100 replications.

### 2.9. Comparative Analysis of Gene Content

Comparison of the gene content between genomes of *B. naardenensis* and *B. bruxelensis* was done using program BLAST 2.2.29+ as described earlier [27]. 

## 3. Results

### 3.1. Genome Structure

The genome of *B. naardenensis* CBS 7540 was assembled into 76 contigs. The details of genome assembly are summarized in Table 1. The total nucleotide content was estimated to be 11,283,072 bp, of which only 752 nucleotide residues could not be unambiguously assigned. Such ambiguous nucleotide positions were restricted to 45 N-regions, which were potentially generated at contigs links sites during scaffolding. Contigs with lengths exceeding 10 kb represented 71% of the final assembly, which is comparable to other assemblies generated by SOAPdenovo1.05 [34]. Sequence length of the shortest contig, which along with the larger contigs, compose half of genome sequence (N50), is 395,348 bp, which suggested high genome contiguity. Furthermore, 10% of the genome assembly consisted of contigs shorter than 87,012 bp. The GC-content of *B. naardenensis* CBS 7540 genome assembly was found to be 44.5%, which is comparable to that of *B. bruxellensis* [34].

### 3.2. Genome Annotation

An advanced annotation approach was used to increase gene prediction accuracy (Figure S1). In total, 5168 putative protein-coding sequences were identified and annotated, which is comparable to that of *B. bruxellensis* CBS 2499 [25]. The annotation details of the *B. naardenensis* CBS 7540 genome are presented in Table 2. Statistics of the CBS 7540 genome annotation are in line with other published *Brettanomyces* yeasts genomes. 

Functional genome annotation of the *B. naardenensis* CBS 7540 genome based on Blast2Go and KOG database is presented is Tables S1 and S2, respectively. The functional annotation is of particular use for future studies on omics analysis in this species. 

### 3.3. Heterozygosity

Analysis of polymorphisms of the CBS 7540 genome was performed by mapping the CBS 7540 reads to the de novo assembly of the CBS 7540 genome. Reads generated by CBS 7540 genome resequencing mapped to assembly sequence of the CBS 7540 genome with 100 x times coverage. We detected 851 variants (Table 3 and Table S3), which is much lower than that in highly dynamic genome of *B. bruxellensis* CBS 11270 [27]. This could indicate a haploid or highly homozygous genome. 

We detected 323 single-nucleotide polymorphisms (SNPs), constituting 0.002% of the genome size. Transitions (i.e., purine–purine or pyrimidine–pyrimidine exchanges) were detected more frequently than transversions (Table 4). 

Most of the heterozygous features were observed in non-coding genome regions; 736 variants, which were detected outside the open reading frames, are summarized in Table S4; 88 variants occurred in coding sequences, and in total, 49 genes with SNPs were identified (Table 5). Only four genes encoding unnamed protein products had 10 variants or more: *DEKNAAT104779* (24 variants), *DEKNAAT104433* (14 variants), *DEKNAAT102160* (12 variants), and *DEKNAAT103651* (10 variants). The corresponding best BLAST hits in *B. bruxellensis* AWRI1499 were a predicted subunit of the CCR4-NOT complex (*AWRI1499_4082*), a predicted iron homeostasis modulator (*AWRI1499_1657*), hypothetical protein (*AWRI1499_4852*), and a predicted CCR4-NOT transcription subunit 3 (*AWRI1499_3655*). An additional 45 genes had between one and nine variants (Table S5).

**Figure 1:**
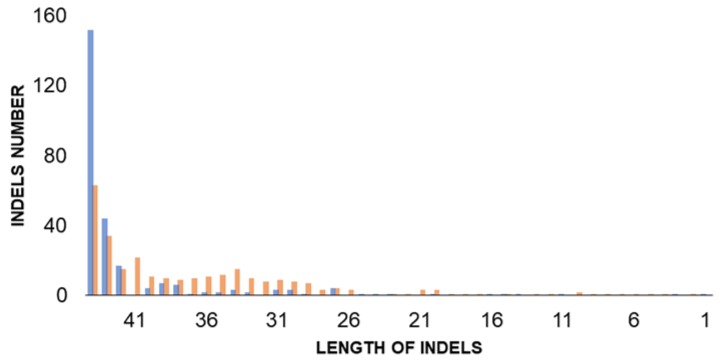
Distribution of indels of different sizes in heterozygous sites in the genome of *B. naardenensis* CBS 7540. Red columns indicate number of insertions, blue columns show deletions.

Moreover, 533 indels were found in the CBS 7540 genome. Micro/mini satellites were observed among some indels (Table S3). Indels varied in size from 1 to 129 nucleotides (Figure 1), which is similar to that in *B. bruxellensis* CBS 11270 [27]. The length of the indels inversely correlated to the frequency. 

### 3.4. Phylogenetic Analysis

**Figure 2:**
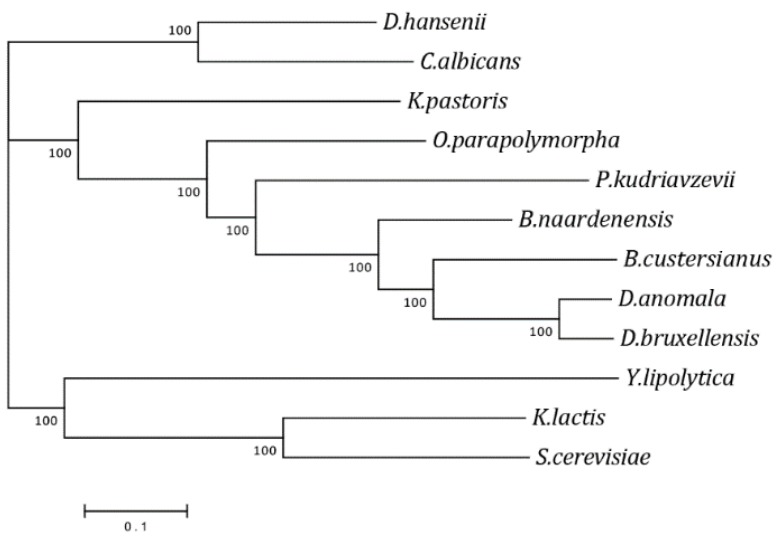
The phylogenetic relationships within yeasts of the genus *Brettanomyces* and other yeast species.

Previous analyses of phylogenetic relationships within the genus *Brettanomyces* have been contradictory [35,36]. The availability of a whole genome sequence of *B. naardenensis* reported here allowed us to perform a phylogenetic analysis based on the concatenation of all gene groups (Figure 2). Our results support previous findings on relationships between the *Brettanomyces* species based on the complete 26S rRNA gene sequence analysis [35]. The wine-spoiling Crabtree-positive species, *B. bruxellensis* and *B. anomala,* are closely related. The Crabtree-negative yeast *B. custersianus* is less related to Crabtree-positive *Brettanomyces* species. *B. naardenensis* is the most distantly related to other *Brettanomyces* species. The last was also suggested by the D1/D2 tree [36].

### 3.5. Genes Associated with Food-Related Traits of B. Naardenensis CBS 7540

#### 3.5.1. Assimilatory Pathways

*B. naardenensis* can utilize a broad range of carbohydrates as carbon sources including galactose, maltose, xylose, trehalose, cellobiose, rhamnose, and arabinose. Sugar components of soft drinks could present a carbon source for *B. naardenensis* growth, resulting in turbidity or formation of microbial sediment in beverage product. 

The CBS 7540 genome included a number of hypothetical genes predicted to be involved in xylose metabolism, including a putative D-xylulose reductase (*DEKNAAC100143*); a putative xylulokinase (*DEKNAAC100346*); a putative xylose/arabinose reductase (*DEKNAAC102931*); and a putative NADPH-dependent D-xylose reductase (*DEKNAAC104786*). It has previously been shown that the CBS 6042 strain of *B. naardenensis* could ferment xylose with 1.8 g/L of ethanol produced from 20 g/L of xylose [37]. Broad substrate utilization by non-conventional yeast species is a useful property for the reduction of residual sugar content in production of super-attenuated and lower-calorie beers [11]. 

Investigation of alternative nitrogen metabolism genes did not result in identification of the gene cluster (*NIT* genes) encoding for the nitrate assimilation pathway: Nitrate transporter (*YNT1*); nitrate reductase (*YNR1*); nitrite reductase (*YNI1*) in the genome of *B. naardenensis* CBS 7540. This is different to some *B. bruxellensis* strains [38] and could confer an advantage of this species over *B. naardenensis* in a nitrate-rich environment such as hopped beer.

*B. naardenensis* can survive in bottled drinks under oxygen-limited conditions. We did not identify a *URA1* gene in *B. naardenensis*. The genome of *B. bruxellensis* was previously shown to contain *URA9* (encoding oxygen-dependent mitochondrial dihydroorotate dehydrogenase (DHOD)) but not *URA1* (oxygen-independent cytoplasmic DHOD) [39]. It remains unclear which alternative mechanism *B. bruxellensis* could use for uracil biosynthesis under anaerobic conditions [40]. This is different in *S. cerevisiae,* which has lost *URA9* and employs *URA1* for uracil synthesis under anaerobic conditions.

#### 3.5.2. Genes Putatively Involved in Production of Volatiles

We have annotated genes of five alcohol dehydrogenases (ADH) in the *B. naardenensis* CBS 7540 genome: *DEKNAAC101096, DEKNAAC103026,* and *DEKNAAC103616*; *ADH3 DEKNAAC105038* and a NADPH-dependent medium-chain *ADH* with broad substrate specificity *DEKNAAC104540*. Similar to *B. bruxellensis*, *B. naardenensis* has several independently duplicated *ADH* and *ADH*-like genes, which are presumably involved in metabolism of alcohols, including ethanol, and also other aromatic compounds [25]. Interestingly, a previous study suggested that in *B. bruxellensis,* one *ADH* gene is involved in both ethanol production and consumption [38], which is similar to respiratory yeasts *Scheffersomyces stipitis* [41] and *Wickerhamomyces anomalus* [42].

The enzyme beta-glucosidase hydrolyzes glucose from glycosides derived from hops or fruit ingredients of beverage forming volatile aglycones. Using blast search versus beta-glucosidase AWRI1499_4190 of *B. bruxellensis* AWRI1499, we identified two beta-glucosidase candidate genes in the genome of *B. naardenensis* CBS 7540: unnamed proteins *DEKNAAC101161* (e-value 0.0) and *DEKNAAC101664* (e-value 2e^−111^). This indicates that *B. naardenensis* has genetic potential for production of flavor-active aglycones. Further metabolic conversions of monoterpenes by complex brewing cultures can contribute to the generation of fruity or floral odors [15].

The absence of *ATF1* and *ATF2* genes involved in production of acetate esters disables *Brettanomyces* from producing the banana-flavored isoamyl acetate and honey-flavored 2-phenylacetate. However, the presence of esterase could potentially enable the formation of ethyl esters with tropic fruit-like odors: Ethyl acetate, hexanoate, and octanoate. Esterase *DEKNAAC103380* was annotated in the genome of *B. naardenensis* CBS 7540. Esterification of cheesy-flavored long-chain fatty acids (C9, C10, C12, C14, C16) by *Brettanomyces* yeasts was suggested to contribute in formation of grape-like beer flavor [15]. Two other unnamed genes, *DEKNAAC104471* and *DEKNAAC100147*, have high sequence similarity to *DEKNAAC103380* and could potentially encode additional esterases.

Both *S. cerevisiae* and *B. bruxellensis* can convert cinnamic acids derived from grape skin into hydroxycinnamic acids using esterase following conversion into hydroxysterene using cinnamate decarboxylase. However, unlike *S. cerevisiae, B. bruxellensis* displays vinyl phenol reductase (VPR) activity, which catalyzes further conversion of hydroxystyrene into a volatile ethyl derivate [6]. Ethyl phenols are characteristic “Brett” off-flavor markers, which are described as “mousy” and represent molecular agents of wine or beer spoilage. VPR reaction was recently shown to be catalyzed by an enzyme with dual superoxide dismutase and NADH-dependent reductase activity [43]. A BLAST search of the *B. naardenensis* CBS 7540 genome with the *B. bruxellensis* ST05.12/26 VPR sequence identified a likely orthologous gene (*DEKNAAC101290*, e-value 5 × 10^−100^), which suggested that this enzyme may play a role in off-odors formation in *B. naardenensis* as well.

Sensation of acetate is a matter for discussion—its presence is desirable for certain brewing styles and in other beverages is perceived as a sign of spoilage. In contrast to *B. bruxellensis,* which was reported as a potent acetate producer, *B. naardenensis* was described to generate moderate amounts of acetate under aerobic conditions [44]. The reason for the acetate overproduction phenotype is thought to be the insufficient activity of the acetyl-CoA synthetase responsible for the conversion of acetate to acetyl-CoA [45,46]. Several aldehyde dehydrogenases, *DEKNAAC103751*, *DEKNAAC103492,* and *DEKNAAC101110*; aldehyde dehydrogenase (NADP+) Ald4 *DEKNAAC100618;* and mitochondrial aldehyde dehydrogenase *DEKNAAC103573* were annotated in the genome of *B. naardenensis* CBS 7540. We identified several *B. naardenensis* CBS 7540 gene candidates for acetyl-CoA synthetase, *DEKNAAC103441, DEKNAAC102074, DEKNAAC102090*.

The inhibition of alcoholic fermentation under anaerobic conditions—commonly known as the Custer effect—characteristic of yeasts belonging to the *Brettanomyces/Dekkera* genus [47] is associated with overproduction of NADH during acetate production. Low expression of NADH oxidizing enzymes of glycerol production pathway in *B. bruxellensis* [48] is not sufficient to provide a sink for NADH. 

Alternative respiration involving salicylhydroxamic acid (SHAM)-sensitive alternative oxidase (AOX) was suggested to present an additional mechanism behind the Custer effect [39]. The hypothetical gene *DEKNAAC101381* likely encodes AOX in *B. naardenensis* CBS 7540 (best BLAST hit is alternative oxidase AWRI1499_1979 with an e value of 10^–162^). 

#### 3.5.3. Putative Stress Tolerance Genes

*B. naardenensis* was shown to survive in pH ranging from 2.6–3.2 [7,8]. The mechanism behind this notable stress tolerance is unclear. At low extracellular pH, undissociated weak acids become able to diffuse into the cytosol. The higher intracellular cytoplasm pH leads to dissociation of weak organic acids, which diminishes growth by the drop in intracellular pH, ATP depletion, intracellular accumulation of anions [49], and inhibition of DNA replication [50]. Plasma membrane ATPase pumps out protons, consuming ATP and thus declining biosynthetic processes. Weak organic acids can cause additional destructive effects. Acetic acid was shown to induce generation of reactive oxygen species (ROS) in the cell [51].

Studies on *S. cerevisiae* determined several genes conferring high acetic acid tolerance: A transcriptional activator involved in the response to weak acid stress, *HAA1; CUP2,* paralog of *HAA1*; glyoxylase gene *GLO1* (an enzyme responsible for the detoxification of methylglyoxal, a side-product of the triose phosphate isomerase reaction in glycolysis); oxidative stress-resistance gene DOT5 encoding nuclear thiol peroxidase, and vacuolar membrane Atpase *VMA7* [52]. The corresponding orthologous genes in the genome of *B. naardenensis* CBS 7540 were identified as *DEKNAAC104025, DEKNAAC102923, DEKNAAC104208,* and *DEKNAAC101910*, respectively.

On other hand, *B. naardenensis* has remarkably low thermotolerance, which could limit its dissemination in human-related habitats. *B. naardenensis* was able to grow at 25 °C but not 30 °C. This is different to *B. bruxellensis*, which can grow at 37 °C [53]. Since data on ethanol tolerance of *B. naardenensis* are scarce, it is unclear if ethanol levels can act as additional limiting factor.

## 4. Discussion

Despite the recent interest in *Brettanomyces* species research, the literature on *B. naardenensis* is rather scarce. This study represents the first genomic investigation of the *B. naardenensis*. Genomes of most *Brettanomyces* species became recently available [19,20,21,22,23,24,25,26,27,28] and now only *Brettanomyces* nanus, which was repeatedly isolated from breweries at different sites in Sweden [47,54,55], remains to be sequenced.

The genome size 11.3 Mbp of *B. naardenensis* CBS 7540 is in the range of that determined for other *Brettanomyces* species [19,20,21,22,23,24,25,26,27,28]. Several strains of *B. naardenensis,* CBS 6040, CBS 6041, CBS 6042, CBS 6107, and CBS 6117, have been isolated from carbonated soft drinks in USA, Belgium, Netherlands, Norway, and Denmark, respectively [44]. CBS 7540 investigated in this study is a strain of African origin. A recent study showed that geographical localization and industrial fermentation environment of origin had similar impacts on the total genetic variance of the *B. bruxellensis* population, suggesting an anthropic influence on the spatial biodiversity of this microorganism [56]. Similar to *B. bruxellensis,* almost no isolates from “natural” non-human related habitats were reported for *B. naardenensis*. All above-mentioned *B. naardenensis* strains were described as soft drink spoilage microorganisms, suggesting notable species tolerance to low pH and osmotic stress. The low number of reports on the occurrence of *B. naardenensis* in wine [9] and beer [6] indicates that the species is less competitive in these environments than *B. bruxellensis, B. anomala,* and *B. custersianus* [57]. A genome hybridization event resulting in highly heterozygous allotriploid genotype of some Australian *B. bruxellensis* wine strains could confer a selective advantage of this species in the presence of the high levels of sulfur dioxide, a preservative used in winemaking [22,23,58]. In the present study, we identified genes involved in tolerance to low pH, which might provide deeper understanding of molecular basis behind occurrence of *B. naardenensis* in soft drinks. 

The recent rise in craft and specialty beer production has awoken an interest in non-conventional yeast species and resulted in more careful consideration of organoleptic properties of fermented beverages flavor [15]. Organoleptic properties of beverages such as turbidity, presence of sediments, acidic taste, and special aroma profiles depends on brewing style and is the matter of personal preferences. We have determined the *B. naardenensis* genes putatively involved in metabolism of alternative carbon sources, which could contribute to production of innovative beverages with high attenuation degree and low alcohol content. A number of identified candidate genes responsible for generation of volatile compounds suggests that further substrate optimizations might allow for suppressing undesirable and enhancing pleasant aromas generated by *B. naardenensis*.

Interestingly, the fermentative lifestyle has evolved in one species of this genus, *B. bruxellensis*, while the other species such as *B. naardenensis* are still respiratory, i.e., they are only forming ethanol under oxygen limitation. In contrast to the *Saccharomyces* line, development of fermentative lifestyle in *B. bruxellensis* was not preceded by a whole genome duplication. There are indications that promoter re-wiring and amplification of fermentative genes such as alcohol dehydrogenase genes played a role in developing the fermentative live style [5]. Those re-organizations of metabolic regulation might have been promoted by a highly dynamic genome of *B. bruxellensis* [27]. In contrast to the highly dynamic genome structure of *B. bruxellensis*, a low number of identified variants points towards high genome stability of *B. naardenensis* CBS 7540. Low genetic polymorphism could limit the physiological plasticity of the species abolishing evolution towards fermentative lifestyle—this is consistent with preservation of respiratory phenotype of *B. naardenensis*. 

The teleomorphic state of *B. naardenensis* [59] was shown to be an artefact caused by the staining method [60]. The high genome stability is rather uncommon for asexual *Brettanomyces* species. A high level of homozygosity between *Saccharomyces* strains was shown to be associated with sporulation and selfing phenomena [61,62]. Phylogenetic analysis showed that *B. naardenensis* diverged from other *Brettanomyces* species earliest, and therefore molecular physiology of this species might be distinct from other species of this genus. 

The genome sequence of *B. naardenensis* provides a basis for further research efforts to understand notable stress tolerance and uncover the full biotechnological potential of this yeast in the production of new-generation beverages.

## 5. Conclusions

Despite frequent isolation of *B. naardenensis* from food-related environments, this species remains poorly investigated. Genome survey of *B. naardenensis* provides the first insights into the genetic landscape of this yeast. We present highly refined RNA-guided genome annotation. Additionally, functional annotations provide tools for further system biology studies on this species. Genome size and gene content of *B. naardenensis* is comparable to other annotated *Brettanomyces* species. However, high genome homozygosity is different from dynamic heterozygous genome of *B. bruxellensis*. Apart from highly diverged DNA and protein sequences between these two *Brettanomyces* species, promotor rewiring boosted differentiation of *B. naardenensis* and *B. bruxellensis* lifestyles into respiratory and fermentative, respectively. Our analysis supports distant phylogenetic relations between these species. Identification of genes involved in tolerance to low pH explains ability of *B. naardenensis* to spoil soft drinks. We have determined *B. naardenensis* genes that could contribute to production of innovative beverages with high attenuation degree and low alcohol content. Candidate genes responsible for generation of volatile compounds were identified, which provides the basis for future exploration the full potential of *B. naardenensis*.

## Figures and Tables

**Table 1:** Details of *B. naardenensis* CBS 7540 genome assembly.

Assembly Characteristic	Description
Number of contigs	76
Number of contigs >1 kb	76
Number of contigs >10 kb	54
N50	395,348 bp
N90	87,012 bp
Number of nucleotides	11,283,072 bp
Number of Ns nucleotides	752 bp
Number of N-regions	45
GC-content	44.5%

**Table 2:** Annotation details of the *B. naardenensis* CBS 7540 genome.

Annotation Feature	Counts
Genes	5168
mRNAs	5181
Exons	5692
Introns	496
Mean intron frequency per gene	0.1
Mean intron length (bp)	146
Mean CDS length (bp)	1488
Mean mRNA length (bp)	1528
Total exon length (bp)	7,840,339
Total intron length (bp)	72,570

**Table 3:** Summary of variants analysis in heterozygous sites of the *B. naardenensis* CBS 7540 genome.

Variant Type	Counts
SNP	323
Indel	533
Total variant	851

**Table 4:** Counts of different types of nucleotide transversions and transitions in heterozygous sites in the genome of *B. naardenensis* CBS 7540.

SNP Type	Counts
A/C	19
A/G	41
T/G	6
T/A	25
C/T	51
T/C	30
C/A	43
G/T	18
A/T	24
C/G	18
G/C	11
G/A	37

**Table 5:** Distribution of genes with different numbers of variants in heterozygous sites in the genome of *B. naardenensis* CBS 7540.

Number of Variants	Gene Counts
24	1
14	1
12	1
10	1
7	1
6	1
5	1
4	1
3	4
2	7
1	30

## Supplementary Materials

The following are available online at https://www.mdpi.com/2076-2607/7/11/489/s1, Figure S1: Genome annotation pipeline, Table S1: Functional Blast2Go annotation of *B. naardenensis* CBS 7540 genome, Table S2: Functional KOG annotation of *B. naardenensis* CBS 7540 genome, Table S3: Variants in CBS 7540, Table S4: Characterization of variants in CBS 7540, Table S5: List of genes in CBS 7540 with variants.
