# Supplementary figures and images for: Assembly and Analysis of the Genome Sequence of the Yeast Brettanomyces naardenensis CBS 7540
Source: Microorganisms. 2019 Oct 26;7(11):489. doi: 10.3390/microorganisms7110489 (PMC6921048; doi:10.3390/microorganisms7110489)

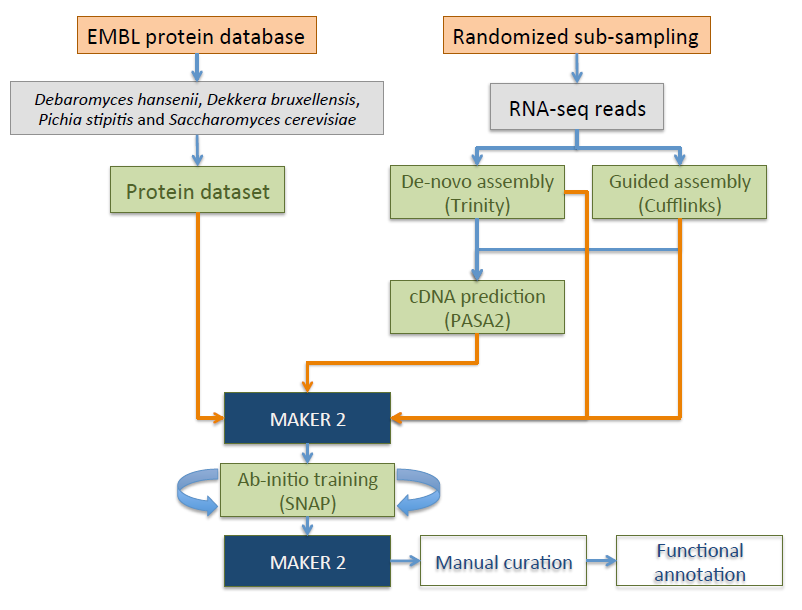

Supplement: Supplementary file 1 [file microorganisms-07-00489-s001.zip › Supplementary_Materials/Figure_S1.docx]
